# Isolation and structure determination of a new analog of polycavernosides from marine *Okeania* sp. cyanobacterium

**DOI:** 10.3762/bjoc.20.57

**Published:** 2024-03-21

**Authors:** Kairi Umeda, Naoaki Kurisawa, Ghulam Jeelani, Tomoyoshi Nozaki, Kiyotake Suenaga, Arihiro Iwasaki

**Affiliations:** 1 Department of Chemistry, Faculty of Science and Technology, Keio University, 3-14-1 Hiyoshi, Kohoku-ku, Yokohama, Kanagawa 223-8522, Japan; 2 Department of Biomedical Chemistry, Graduate School of Medicine, The University of Tokyo, 7-3-1 Hongo, Bunkyo-ku, Tokyo 113-0033, Japan; 3 Department of Applied Chemistry, Faculty of Science and Engineering, Chuo University, 1-13-27 Kasuga, Bunkyo-ku, Tokyo 112-8551, Japan

**Keywords:** macrolide glycoside, marine cyanobacterium, marine natural products, polycavernosides, terminal alkyne

## Abstract

Polycavernoside E (**1**), a new polycavernoside analog, was isolated from a marine *Okeania* sp. cyanobacterium. The relative configuration was elucidated primarily by analyzing the two dimensional nuclear magnetism resonance (2D NMR) data. The absolute configuration was clarified by comparing the electronic circular dichroism (ECD) data of **1** with those of known analogs. Polycavernoside E (**1**) exhibited moderate antitrypanosomal activity against *Trypanosoma brucei rhodesiense*. Furthermore, the isolation of polycavernoside E (**1**) from marine cyanobacteria provides additional evidence that marine cyanobacteria, and not red algae, are responsible for the biosynthesis of polycavernosides.

## Introduction

**Figure 1:**
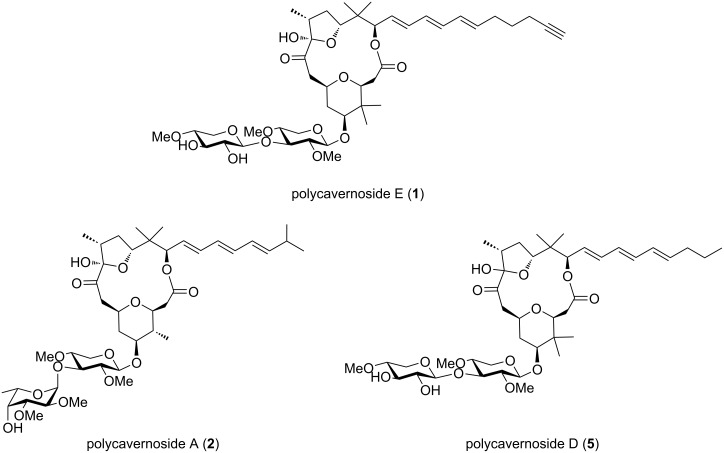
Structures of compounds **1**, **2**, and **5**.

In 1991, an outbreak of food poisoning caused by a species of red algae known as ‘*Polycavernosa tsudai*’ occurred in Guam, which resulted in killing of three people. Two novel macrolide glycosides, polycavernosides A (**2**) and B (**3**), were reported as the causative compounds for the illness [1]. After that, the second fatal food poisoning incidents occurred in the Philippines caused by the ingestion of polycavernoside A (**2**)-contaminated red algae [2]. Subsequently, polycavernoside analogs such as polycavernoside C (**4**) were isolated from red algae [3–4]. In 2015, Navarro et al. isolated polycavernoside D (**5**) from a marine *Okeania* sp. cyanobacterium [5]. They suggested that polycavernosides were produced by marine cyanobacteria based on their high content and structural similarity to other cyanobacterial metabolites. In this study, polycavernoside E (**1**), a new polycavernoside analog, was isolated from a marine *Okeania* sp. cyanobacterium obtained from Okinawa Prefecture, Japan (Figure 1). This finding provides additional evidence that polycavernosides are secondary metabolites derived from marine *Okeania* sp. cyanobacteria.

## Results and Discussion

The EtOH extract of marine *Okeania* sp. cyanobacterium (340 g, wet weight) collected from Akuna Beach, Okinawa, Japan, was partitioned between EtOAc and H_2_O. The EtOAc fraction was further partitioned into 90% aqueous MeOH and hexane. The aqueous MeOH portion was purified by reversed-phase column chromatography (ODS silica gel, MeOH/H_2_O), automated flash chromatography (hexane/EtOAc), and repeated reversed-phase HPLC to give polycavernoside E (**1**, 0.5 mg as a colorless oil). The isolation of compound **1** was directed by its characteristic UV absorption around 270 nm.

**Figure 2:**
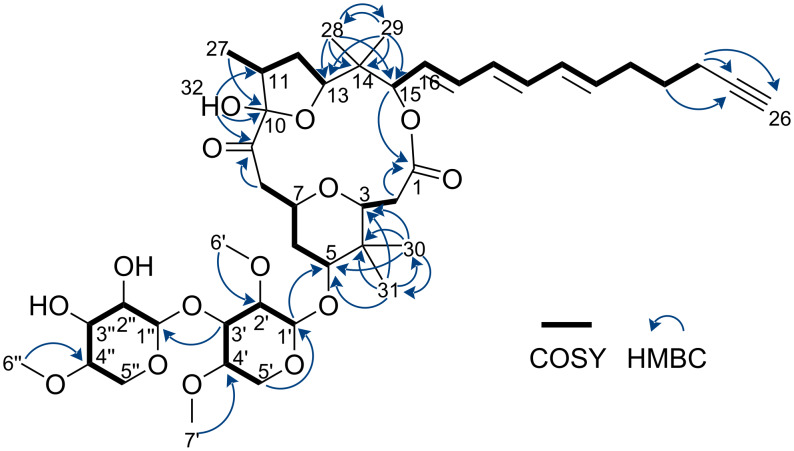
Planar structure of polycavernoside E (**1**) based on 2D NMR analysis.

The molecular formula of **1** was determined to be C_44_H_66_O_15_ based on the HRESIMS data. The NMR data for **1** are summarized in Table 1. The ^1^H NMR spectrum of compound **1** was similar to those of known polycavernosides but matched none of them, suggesting that **1** was a new analog of polycavernosides [1,3–5]. A detailed analysis of the NMR data revealed the planar structure of **1**, as shown in Figure 2. COSY and HMQC spectral analyses revealed several partial structures, indicated by the bold bonds in Figure 2. Four HMBC were observed from singlet methyl signals: δ_H_ 0.85 (H-28)/δ_C_ 19.4 (C-29), δ_H_ 0.86 (H-29)/δ_C_ 17.8 (C-28), δ_H_ 0.94 (H-30)/δ_C_ 13.9 (C-31), and δ_H_ 0.90 (H-31)/δ_C_ 22.2 (C-30). These correlations elucidated the presence of two *gem*-dimethyl groups. Moreover, three HMBC, δ_H_ 4.03 (H-5a’)/δ_C_ 106.1 (C-1’), δ_H_ 3.61 (H-6’)/δ_C_ 83.8 (C-2’), and δ_H_ 3.45 (H-7’)/δ_C_ 78.5 (C-4’), revealed the presence of a 2,4-di-*O*-methylpyranose substructure. Furthermore, an HMBC, δ_H_ 3.48 (H-6”)/δ_C_ 78.7 (C-4”), along with typical chemical shifts and coupling constants from C-1” to C-5” obtained in CD_3_OD (Table 2), indicated the presence of a 4-*O*-methylpyranose substructure. The HMBC, δ_H_ 3.64 (H-3’)/δ_C_ 103.0 (C-1”), indicated that these two sugar structures were connected through a glycosidic bond.

**Table 1:** NMR data for polycavernoside E (**1**) in CDCl_3_.

position	δ_C_, type^a^	δ_H_^b^ (*J* in Hz)	COSY	selected HMBC

1	171.9, C			
2	35.6, CH_2_	2.29, m	3	1
3	82.0, CH	3.43, m	2	
4	38.3, C			
5	85.3, CH	3.32, m	6a, 6b	
6a	37.7, CH_2_	1.95, m	5, 6b, 7	
6b		1.61, m	5, 6a, 7	
7	83.8, CH	3.07, m	6a, 6b, 8a, 8b	
8a	42.1, CH_2_	3.08, m	7, 8b	9
8b		2.00, m	7, 8a	
9	206.9, C			
10	103.0, C			
11	39.7, CH	2.74, m	12a, 12b, 27	
12a	33.6, CH_2_	2.01, m	11, 12b, 13	
12b		1.70, m	11, 12a	
13	83.5, CH	4.18, dd (11.3, 5.0)	12a, 12b	
14	39.8, C			
15	78.4, CH	5.17, d (8.2)	16	1
16	127.4, CH	5.55, dd (8.2, 15.0)	15, 17	
17	135.4, CH	6.26, m	16, 18	
18	130.1, CH	6.09, m	17, 19	
19	133.9, CH	6.13, m	18, 20	
20	131.2, CH	6.08, m	19, 21	
21	134.6, CH	5.67, dt (15.0, 7.3)	20, 22	
22	31.8, CH_2_	2.19, m	21, 23	
23	28.1, CH_2_	1.62, m	22, 24	25
24	17.9, CH_2_	2.18, m	23, 26	25, 26
25	84.6, C			
26	68.6, CH	1.95, t (2.7)	24	
27	13.3, CH_3_	0.99, d (6.8)	11	10
28	17.8, CH_3_	0.85, s		13, 14, 15, 29
29	19.4, CH_3_	0.86, s		13, 14, 15, 28
30	22.2, CH_3_	0.94, s		3, 4, 5, 31
31	13.9, CH_3_	0.90, s		3, 4, 5, 30
32	OH	4.47, s		9, 10, 11
1’	106.1, CH	4.27, d (7.7)	2’	5
2’	83.8, CH	3.07, m	1’, 3’	
3’	79.9, CH	3.64, m	2’, 4’	1”
4’	78.5, CH	3.27, m	3’, 5a’, 5b’	
5a’	63.2, CH_2_	4.03, dd (11.3, 5.0)	4’, 5b’	1’
5b’		3.12, m	4’,5a’	
6’	61.1, CH_3_	3.61, s		2’
7’	58.8, CH_3_	3.45, s		4’
1”	103.0, CH	4.87, d (4.5)	2”	
2”	71.7, CH	3.53, m	1”, 3”	
3”	71.0, CH	3.75, m	2”, 4”	
4”	78.7, CH	3.34, m	3”, 5a”, 5b”	
5a”	60.1, CH_2_	4.23, dd (12.2, 3.2)	5b”, 4”	
5b”		3.46, m	5a”, 4”	
6”	58.1, CH_3_	3.48, s		4”
7”	-	OH		
8”	-	OH		

^a^Measured at 400 MHz. ^b^Measured at 100 MHz.

The geometries of the two olefins at C-16 and C-20 were determined to be *trans* based on the large coupling constants, ^3^*J*_H-16/H-17_ 15.0 Hz and ^3^*J*_H-20/H-21_ 15.0 Hz, respectively. The geometry of the remaining double bond at C-18 was established to be *trans* by comparing the ^13^C NMR chemical shifts at C-16 and C-21 between **1** and polycavernoside D (**5**) (Table S1 in Supporting Information File 1) [5]. In addition, a ^4^*J* long-range coupling between δ_H_ 1.95 (H-26) and δ_H_ 2.18 (H-24) and three HMBC δ_H_ 1.62 (H-23)/δ_C_ 84.6 (C-25), δ_H_ 2.18 (H-24)/δ_C_ 84.6 (C-25), and δ_H_ 2.18 (H-24)/δ_C_ 68.6 (C-26) revealed a terminal alkyne structure. Additionally, COSY correlations shown in Figure 2 revealed the side chain structure of **1** containing a terminal alkyne and a conjugated *trans* triene (C-15 to C-26).

We then focused on the macrolide structure of **1**. Six HMBC, δ_H_ 0.94 (H-30)/δ_C_ 82.0 (C-3), δ_H_ 0.94 (H-30)/δ_C_ 38.3 (C-4), δ_H_ 0.94 (H-30)/δ_C_ 85.3 (C-5), δ_H_ 0.90 (H-31)/δ_C_ 82.0 (C-3), δ_H_ 0.90 (H-31)/δ_C_ 38.3 (C-4), and δ_H_ 0.90 (H-31)/δ_C_ 85.3 (C-5), along with COSY correlations shown in Figure 2, revealed a chain structure from C-2 to C-8. In addition, eight HMBC, δ_H_ 5.17 (H-15)/δ_C_ 171.9 (C-1), δ_H_ 2.29 (H-2)/δ_C_ 171.9 (C-1), δ_H_ 0.85 (H-28)/δ_C_ 83.5 (C-13), δ_H_ 0.85 (H-28)/δ_C_ 39.8 (C-14), δ_H_ 0.85 (H-28)/δ_C_ 78.4 (C-15), δ_H_ 0.86 (H-29)/δ_C_ 83.5 (C-13), δ_H_ 0.86 (H-29)/δ_C_ 39.8 (C-14), and δ_H_ 0.86 (H-29)/δ_C_ 78.4 (C-15), and COSY correlations shown in Figure 2, clarified the connection of C-1 to C-8 and C-15 to C-11(-C27) through an ester bond. Furthermore, five HMBC, δ_H_ 0.99 (H-27)/δ_C_ 103.0 (C-10), δ_H_ 4.47 (H-32)/δ_C_ 206.9 (C-9), δ_H_ 4.47 (H-32)/δ_C_ 103.0 (C-10), δ_H_ 4.47 (H-32)/δ_C_ 39.7 (C-11), and δ_H_ 3.08 (H-8a)/δ_C_ 206.9 (C-9) connected C-11 and C-8 through a ketone carbonyl carbon at C-9 and hemiacetal carbon at C-10, revealing the 16-membered macrolide structure of **1**. The HMBC, δ_H_ 4.27 (H-1’)/δ_C_ 85.3 (C-5), revealed that the disaccharide moiety was connected to C-5. Finally, considering the molecular formula of **1** and the chemical shifts of known polycavernosides, we established the presence of a THP ring containing C-3 to C-7 and a THF ring containing C-10 to C-13 in the macrolide structure. Consequently, we established the planar structure of **1**, as shown in Figure 2.

**Figure 3:**
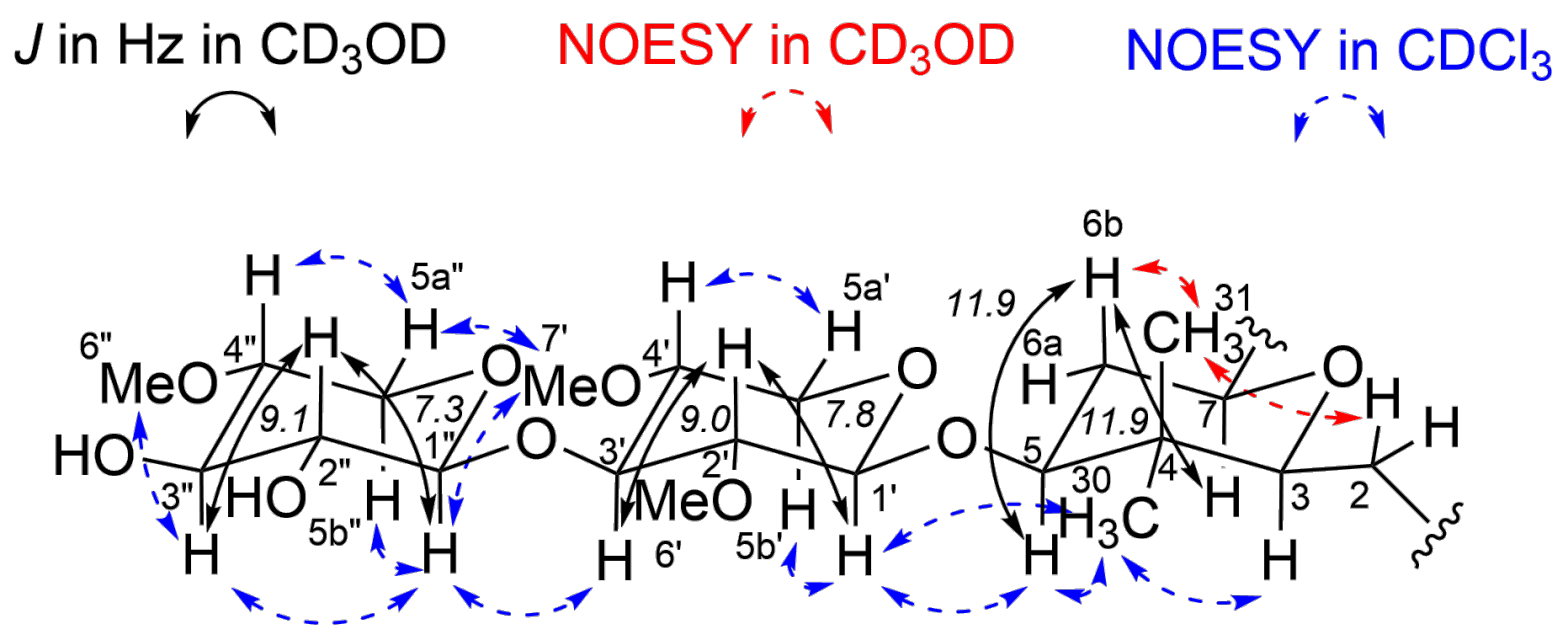
Relative configuration of the THP ring and disaccharide moiety of **1**.

The relative configuration of compound **1** was determined based on the NMR data obtained in CD_3_OD and CDCl_3_ (Table 1 and Table 2). The relative configuration of the THP ring and the disaccharide moiety of **1** was determined by analyzing the proton coupling constants and NOESY correlations (Figure 3). The two coupling constants in CD_3_OD, ^3^*J*_H-5/H-6b_ (11.9 Hz) and ^3^*J*_H-6b/H-7_ (11.9 Hz), indicated that H-5, H-6b, and H-7 were in the axial position. The two NOESY correlations in CD_3_OD, δ_H_ 1.80 (H-6b)/δ_H_ 0.91 (H-31) and δ_H_ 2.36 (H-2)/δ_H_ 0.91 (H-31), revealed that H-6b, C-31, and C-2 were located in the same face of the THP ring as shown in Figure 3. Consequently, the relative configuration of the THP ring was determined to be 3S*,5S*,7S*. For the 2,4-di-*O*-methylpyranose moiety, the two large coupling constants in CD_3_OD, ^3^*J*_H-1’/H-2’_ (7.8 Hz) and ^3^*J*_H-2’/H-3’_ (9.0 Hz), indicated that H-1’, H-2’, and H-3’ were in the axial position. The two NOESY correlations in CDCl_3_, δ_H_ 3.45 (H-7’)/δ_H_ 4.87 (H-1”) and δ_H_ 3.45 (H-7’)/δ_H_ 4.23 (H-5a”), revealed that the methoxy group at C-4’ was in the equatorial position and H-4’ was in the axial position. The 2,4-di-*O*-methylpyranose moiety was identified as 2,4-di-*O*-methylxylose. For the 4-*O*-methylpyranose moiety, the two large coupling constants in CD_3_OD, ^3^*J*_H-1”/H-2”_ (7.3 Hz) and ^3^*J*_H-2”/H-3”_ (9.1 Hz), indicated that H-1”, H-2”, and H-3” were in the axial position. NOESY correlations in CDCl_3_, δ_H_ 3.75 (H-3”)/δ_H_ 3.48 (H-6”), revealed that the methoxy group at C-4” was in the equatorial position and H-4” was in the axial position. The 4-*O*-methylpyranose moiety was identified as 4-*O*-methylxylose. The relationship between the relative configuration of the 2,4-di-*O*-methylxylose moiety and 4-*O*-methylxylose moiety was identified using three NOESY correlations in CDCl_3_, δ_H_ 3.45 (H-7’)/δ_H_ 4.87 (H-1”), δ_H_ 3.45 (H-7’)/δ_H_ 4.23 (H-5a”), and δ_H_ 3.64 (H-3’)/δ_H_ 4.87 (H-1”), as shown in Figure 3. Furthermore, the relationship of the relative configuration between the disaccharide moiety and the THP ring was revealed by two NOESY correlations in CDCl_3_, δ_H_ 4.27 (H-1’)/δ_H_ 3.32 (H-5) and δ_H_ 4.27 (H-1’)/δ_H_ 0.94 (H-30), shown in Figure 3. The validity of the relative configurations shown in Figure 3 is further substantiated by the good agreement with the corresponding chemical shifts of polycavernoside D (**5**), both of which possess the same disaccharide moiety attached to a THP ring (Tables S1 and S2 in Supporting Information File 1) [5].

**Table 2:** NMR data for polycavernoside E (**1**) in CD_3_OD.

position	δ_C_, type^a^	δ_H_^b^ (*J* in Hz)	COSY	selected HMBC

1	174.3, C			
2	36.5, CH_2_	2.36, d (7.7)	3	1
3	83.2, CH	3.40, m	2	
4	39.4, C			
5	86.1, CH	3.40, m	6a, 6b	
6a	38.0, CH_2_	1.91, m	5, 6b, 7	
6b		1.80, ddd (11.9, 11.9, 11.9)	5, 6a, 7	
7	76.6, CH	3.65, m	6a, 6b, 8a, 8b	
8a	42.3, CH_2_	2.85, m	7, 8b	9
8b		2.37, m	7, 8a	
9	207.4, C			
10	104.8, C			
11	39.7, CH	2.82, m	12a, 12b, 27	
12a	34.6, CH_2_	1.98, m	11, 12b, 13	
12b		1.62, m	11, 12a	
13	83.8, CH	4.12, dd (11.6, 4.7)	12a, 12b	
14	40.6, C			
15	80.3, CH	5.10, d (8.1)	16	1
16	128.5, CH	5.61, dd (8.1, 15.1)	15, 17	18
17	136.4, CH	6.21, m	16, 18	
18	131.1, CH	6.13, m	17	
19	135.0, CH	6.19, m		
20	132.4, CH	6.12, m	21	
21	135.6, CH	5.72, dt (15.3, 7.1)	20, 22	19
22	32.7, CH_2_	2.22, m	21, 23	
23	29.3, CH_2_	1.60, quint (7.2)	22, 24	25
24	18.5, CH_2_	2.17, m	23	25, 26
25	84.7, C			
26	69.8, CH	2.21, m		
27	13.6, CH_3_	0.98, d (6.8)	11	10
28	18.0, CH_3_	0.868, s		13, 14, 15, 29
29	19.5, CH_3_	0.870, s		13, 14, 15, 28
30	22.3, CH_3_	0.98, s		3, 4, 5, 31
31	14.1, CH_3_	0.91, s		3, 4, 5, 30
1’	107.1, CH	4.33, d (7.8)	2’	5
2’	85.5, CH	3.08, dd (7.8, 9.0)	1’, 3’	
3’	81.9, CH	3.63, m	2’, 4’	1”
4’	79.4, CH	3.27, m	3’, 5a’, 5b’	
5a’	64.3, CH_2_	3.96, dd (11.5, 5.1)	4’, 5b’	1’
5b’		3.16, m	4’, 5a’	
6’	61.3, CH_3_	3.61, s		2’
7’	59.5, CH_3_	3.46, s		4’
1”	105.0, CH	4.62, d (7.3)	2”	
2”	75.3, CH	3.21, dd (7.3, 9.1)	1”, 3”	
3”	76.5, CH	3.39, m	2”, 4”	
4”	81.0, CH	3.18, m	3”, 5a”, 5b”	
5a”	64.1, CH_2_	4.07, dd (11.0, 4.4)	5b”, 4”	
5b”		3.13, m	5a”, 4”	
6”	58.9, CH_3_	3.47, s		4”

^a^Measured at 400 MHz. ^b^Measured at 100 MHz.

The remaining relative configuration of **1** was determined by a comparison of the carbon chemical shifts between **1** and **5** in CDCl_3_ [5]. As shown in Table S1 (Supporting Information File 1), the two sets of data were in good agreement, indicating that the relative configurations of compounds **1** and **5** were identical.

**Figure 4:**
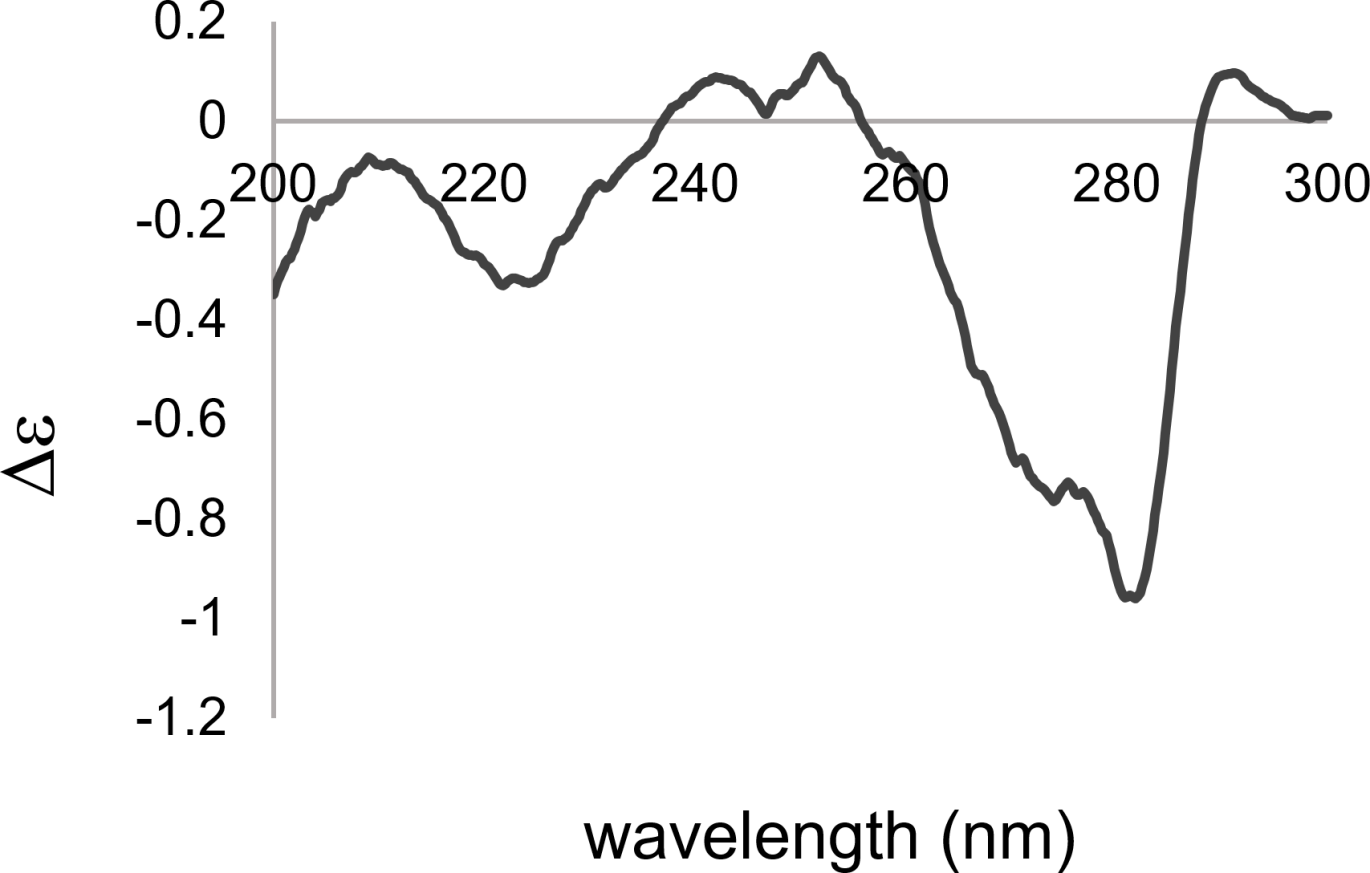
ECD spectrum of **1** in MeOH.

To reveal the absolute configuration, we recorded the ECD spectrum of **1** (Figure 4) and compared it with those of **2** and **5** reported in previous papers [5–6]. We detected a Cotton effect of negative sign at around 280 nm corresponding to the n–π* transition of a ketone group as same as the literature data for **2** and **5**. As a result, the absolute configuration of polycavernoside E was determined to be **1**.

Next, we examined the antitrypanosomal activity of **1** against the bloodstream form of *Trypanosoma brucei rhodesiense* IL-1501 (the causative organism of African trypanosomiasis) (Table 3). As a result, **1** showed moderate growth-inhibitory activities against *Trypanosoma brucei rhodesiense* (IC_50_: 9.9 μM). In addition, we examined the growth-inhibitory activity of **1** on normal human fibroblasts WI-38 (Table 3). In summary, **1** showed a moderate activity against *Trypanosoma brucei rhodesiense*.

**Table 3:** Growth-inhibitory activities of polycavernoside E (**1**).

Compounds	IC_50_ values (μM)	
	
	*T. b. rhodesiense*	WI-38 cells

polycavernoside E (**1**)	9.9 ± 1.5	27
pentamidine^a^	0.006 ± 0.002	–

^a^Positive control.

## Conclusion

In conclusion, we isolated a new polycavernoside analog, named polycavernoside E (**1**), from a marine *Okeania* sp. cyanobacterium. The relative configuration was elucidated mainly by analyzing the 2D NMR data. The absolute configuration was determined based on a comparison of the ECD data for **1** and its known analogs. Polycavernoside E (**1**) showed selective antitrypanosomal activity against *Trypanosoma brucei rhodesiense* with an IC_50_ value of 9.9 μM. This discovery provides additional evidence that polycavernosides, previously thought to be derived from red algae, are produced by the marine *Okeania* sp. cyanobacterium. In the field, this type of cyanobacterium that produces the analog of human lethal toxin is often observed with macroalgae and shells, and, therefore, it can be a potential risk for food poisoning of fishery resources.

## Experimental

### General experimental procedures

Optical rotations were measured with a JASCO DIP-1000 polarimeter. UV spectra were recorded on a UV-3600. ECD spectra were measured with JASCO J-1100. IR spectra were recorded on a Bruker ALPHA instrument. All NMR data were recorded on a JEOL ECX-400/ECS-400 spectrometer for ^1^H (400 MHz) and ^13^C (100 MHz). ^1^H NMR chemical shifts (referenced to the residual solvent signal of CHD_2_OD: δ 3.31, CHCl_3_: δ 7.26) were assigned using a combination of data from COSY and HMQC experiments. Similarly, ^13^C NMR chemical shifts (referenced to the solvent signal CD_3_OD: δ 49.0, CDCl_3_: δ 77.16) were assigned based on HMBC and HMQC experiments. HRESIMS spectra were obtained on a Bruker timsTOF mass spectrometer. For reversed-phase column chromatography, ODS silica gel Cosmosil 75C_18_-OPN (Nacalai Tesque) was used. For medium pressure column chromatography, AFCS (Smart Flash AKROS, Yamazen) consisting of a pump and a UV detector was used. HPLC analysis was conducted using a pump (model PU-2080, Jasco) and a UV detector (model UV-2075, Jasco). All chemicals and solvents used in this study were the best grade available and obtained from a commercial source (Nacalai Tesque).

### Collection and identification of the sample

The marine cyanobacterium producing polycavernoside E (**1**) was collected in March 2022 at the coast in Akuna beach, Yonashiromiyagi, Uruma city, Okinawa, Japan. It was classified into *Okeania* sp. based on the phylogenetic analysis as described in the previous paper (accession no. LC771053) [7].

### Isolation of polycavernoside E (**1**)

In a manner analogous to [7], the collected cyanobacterium (340 g) was extracted with EtOH (0.5 L) for 10 days at room temperature (rt). The extract was filtered, and the residue was homogenized with a blender and re-extracted with EtOH (0.5 L) at room temperature for one day. The extract was filtered, and the combined filtrates were concentrated. The residue was partitioned between EtOAc (3 × 300 mL) and H_2_O (300 mL). The combined organic layers were concentrated, and the residue was partitioned between 90% aqueous MeOH (300 mL) and hexane (3 × 300 mL). The aqueous MeOH layer was concentrated, and the obtained residue (673 mg) was separated by column chromatography on ODS (7 g) eluted with 35 mL of 40%, 60%, 80%, and 90% aqueous MeOH, followed by 35 mL of MeOH and 70 mL of CHCl_3_/MeOH 1:1. The fraction (244.4 mg) eluted with 80% MeOH was subjected to AFCS [Ø 11 × 300 mm; flow rate 5 mL/min; detection at 254 nm; solvent gradient condition, hexane/EtOAc 28:72 → 7:93] to give a fraction that contained compound **1** (17.5 mg, *t*_R_ = 32.0 min). The fraction that contained **1** was further purified by HPLC [Cosmosil 5C_18_-MS-II (Ø 20 mm × 250 mm); solvent MeOH/H_2_O 85:15; flow rate 5 mL/min; detection UV 215 nm] to give a fraction that contained **1** (8.6 mg, last collected fraction). The fraction that contained **1** was further separated by HPLC [Cosmosil Cholester (Ø 20 mm × 250 mm); solvent MeCN/H_2_O 75:25; flow rate 5 mL/min; detection UV 254 nm] to give a fraction that contained **1** (0.6 mg, *t*_R_ = 32.8 min). The fraction that contained **1** was further separated by HPLC [Cosmosil 5PE-MS (Ø 20 mm × 250 mm); solvent MeOH/H_2_O 85:15; flow rate 5 mL/min; detection UV 270 nm] to give **1** (0.5 mg, *t*_R_ = 37.3 min).

Polycavernoside E (**1**): colorless oil; [α]_D_^26^ −19 (*c* 0.04, MeOH); UV (MeOH) λ_max_, nm (log ε): 281 (2.27), 270 (2.96), 260 (2.40); ECD (100 μg/mL; MeOH), λ_max_, nm (Δε): 226 (−0.31), 274 (−0.76), 282 (−0.96); IR (neat): 3443, 2965, 2925, 2896, 1646, 1457, 1086 cm^−1^; HRESIMS (*m*/*z*): [M + Na]^+^ calcd for C_44_H_66_O_15_Na^+^, 857.4294; found, 857.4294.

### In vitro antitrypansomal assay

The bloodstream form of *Trypanosoma brucei rhodesiense* strain IL-1501 was cultured at 37 °C under a humidified 5% CO_2_ atmosphere in HMI-9 medium supplemented with 10% heat-inactivated fetal bovine serum (FBS) [8–9]. For in vitro studies, compounds were dissolved in DMSO and diluted in culture medium prior to being assayed. The maximum DMSO concentration in the in vitro assays was 1%. The compounds were tested in an AlamarBlue serial drug dilution assay to determine the 50% inhibitory concentrations (IC_50_) [10]. Serial drug dilutions were prepared in 96-well microtiter plates, containing 50 μL of culture medium. Subsequently, 50 μL of a parasite suspension with a concentration of 4.0 × 10^4^ cells/mL was introduced into each well. Cultures were incubated for 69 h at 37 °C under a humidified 5% CO_2_ atmosphere. After this time, 10 μL of resazurin (12.5 mg resazurin (Sigma) dissolved in 100 mL phosphate-buffered saline) was added to each well. The plates were incubated for an additional 3 h. The plates were read in a SpectraMax Gemini XS microplate fluorescence scanner (Molecular Devices) using an excitation wavelength of 536 nm and an emission wavelength of 588 nm.

### WI-38 cells assay

In a manner analogous to [7], WI-38 cells were cultured at 37 °C with 5% CO_2_ in DMEM (Nissui) supplemented with 10% heat-inactivated FBS, 100 units/mL penicillin, 100 μg/mL streptomycin, 0.25 μg/mL amphotericin, 300 μg/mL ʟ-glutamine, and 2.25 mg/mL NaHCO_3_. Cells were seeded at 4 × 10^3^ cells/well in 96-well plates (Iwaki) and cultured overnight. Various concentrations of compounds were then added, and cells were incubated for 72 h. Cell proliferation was measured by the MTT assay.

## Supporting Information

File 1 NMR data for polycavernoside E (**1**).

## Data Availability

All data that supports the findings of this study is available in the published article and/or the supporting information to this article.
