# Supplementary material for: Isolation and structure determination of a new analog of polycavernosides from marine Okeania sp. cyanobacterium
Source: Beilstein J Org Chem. 2024 Mar 21;20:645–52. doi: 10.3762/bjoc.20.57 (PMC10964031; doi:10.3762/bjoc.20.57)

BEILSTEIN JOURNAL OF ORGANIC CHEMISTRY

Supporting Information

for

Isolation and structure determination of a new analog of polycavernosides from marine *Okeania* sp. cyanobacterium

Kairi Umeda, Naoaki Kurisawa, Ghulam Jeelani, Tomoyoshi Nozaki, Kiyotake Suenaga and Arihiro Iwasaki

Beilstein J. Org. Chem. **2024,** *20,* 645–652. doi:10.3762/bjoc.20.57

NMR data for polycavernoside E (1)

License and Terms: This is a supporting information file under the terms of the Creative Commons Attribution License (https://creativecommons.org/licenses/by/4.0). Please note that the reuse, redistribution and reproduction in particular requires that the author(s) and source are credited and that individual graphics may be subject to special legal provisions.
The license is subject to the *Beilstein Journal of Organic Chemistry* terms and conditions: (https://www.beilstein-journals.org/bjoc/terms)

Table of contents

Comparison of the carbon chemical shifts between **1** and **5** in $CDCl_3$ (Table S1) S2

Comparison of the proton chemical shifts of disaccharide moiety between **1** and **5** in $CDCl_3$ (Table S2) S3

NMR spectra of polycavernoside E (**1**) in $CDCl_3$ S4

NMR spectra of polycavernoside E (**1**) in CD_3OD S7

Table S1: Comparison of the carbon chemical shifts between polycavernosides E (**1**) and D (**5**) in $CDCl_3$.

position[1]	polycavernoside E (**1**)[2]	polycavernoside D (**5**)[3]
1	171.9, C	171.5, C
2	35.6, CH_2	35.5, CH_2
3	82.0, CH	81.9, CH
4	38.3, C	38.2, C
5[4]	85.3, CH	75.1, CH
6	37.7, CH_2	37.5, CH_2
7	83.8, CH	85.1, CH
8	42.1, CH_2	42.0, CH_2
9	206.9, C	206.5, C
10	103.0, C	102.6, C
11	39.7, CH	39.5, CH
12	33.6, CH_2	33.5, CH_2
13	83.5, CH	83.3, CH
14	39.8, C	39.7, C
15	78.4, CH	78.3, CH
16	127.4, CH	126.9, CH
17	135.4, CH	135.5, CH
18	130.1, CH	129.6, CH
19	133.9, CH	134.2, CH
20	131.2, CH	130.2, CH
21	134.6, CH	136.2, CH
27	13.3, CH_3	13.7, CH_3
28	17.8, CH_3	17.6, CH_3
29	19.4, CH_3	19.2, CH_3
30	22.2, CH_3	22.0, CH_3
31	13.9, CH_3	13.7, CH_3
1’	106.1, CH	106.0, CH
2’	83.8, CH	83.7, CH
3’	79.9, CH	79.9, CH
4’	78.5, CH	78.4, CH
5’	63.2, CH_2	63.1, CH_2
6’	61.1, CH_3	60.9, CH_3
7’	58.8, CH_3	58.6, CH_3
1”	103.0, CH	102.9, CH
2”	71.7, CH	71.6, CH
3”	71.0, CH	71.0, CH
4”	78.7, CH	78.7, CH
5”	60.1, CH_2	60.0, CH_2
6”	58.1, CH_3	58.0, CH_3

[1] The data from C-22 to C-26 were not included as they were useless for the discussion the relative configuration.

[2] Measured at 100 MHz.

[3] Measured at 151 MHz. Navarro, G.; Cummings, M. E.; Lee, J.; Moss, N.; Glukhov, E.; Valeriote, F. A.; Gerwick, L.; Gerwick, W. H. *Environ. Sci. Technol. Lett.* **2015**, *2*, 166-170.

[4] The chemical shift difference was big possibly due to the misassignment of the C-5 carbon chemical shift of **5**.

Table S2: Comparison of the proton chemical shifts of disaccharide moiety between **1** and **5** in $CDCl_3$.

position	polycavernoside E (**1**)[1]	polycavernoside D (**5**)[2]
1’	4.27, CH	4.27, CH
2’	3.07, CH_2	3.08, CH_2
3’	3.64, CH	3.64, CH
4’	3.27, CH	3.27, CH
5a’	4.03, CH_2	4.03, CH_2
5b’	3.12	3.12
6’	3.61, CH_3	3.61, CH_3
7’	3.45, CH_3	3.45, CH_3
1”	4.87, CH	4.86, CH
2”	3.53, CH	3.52, CH
3”	3.75, CH	3.74, CH
4”	3.34, CH	3.35, CH
5a”	4.23, CH_2	4.23, CH_2
5b”	3.46	3.46
6”	3.48, CH_3	3.48, CH_3

[1] Measured at 400 MHz.

[2] Measured at 600 MHz. Navarro, G.; Cummings, M. E.; Lee, J.; Moss, N.; Glukhov, E.; Valeriote, F. A.; Gerwick, L.; Gerwick, W. H. *Environ. Sci. Technol. Lett.* **2015**, *2*, 166-170.

^{1}H NMR (400 MHz, $CDCl_3$) spectrum of polycavernoside E (**1**)

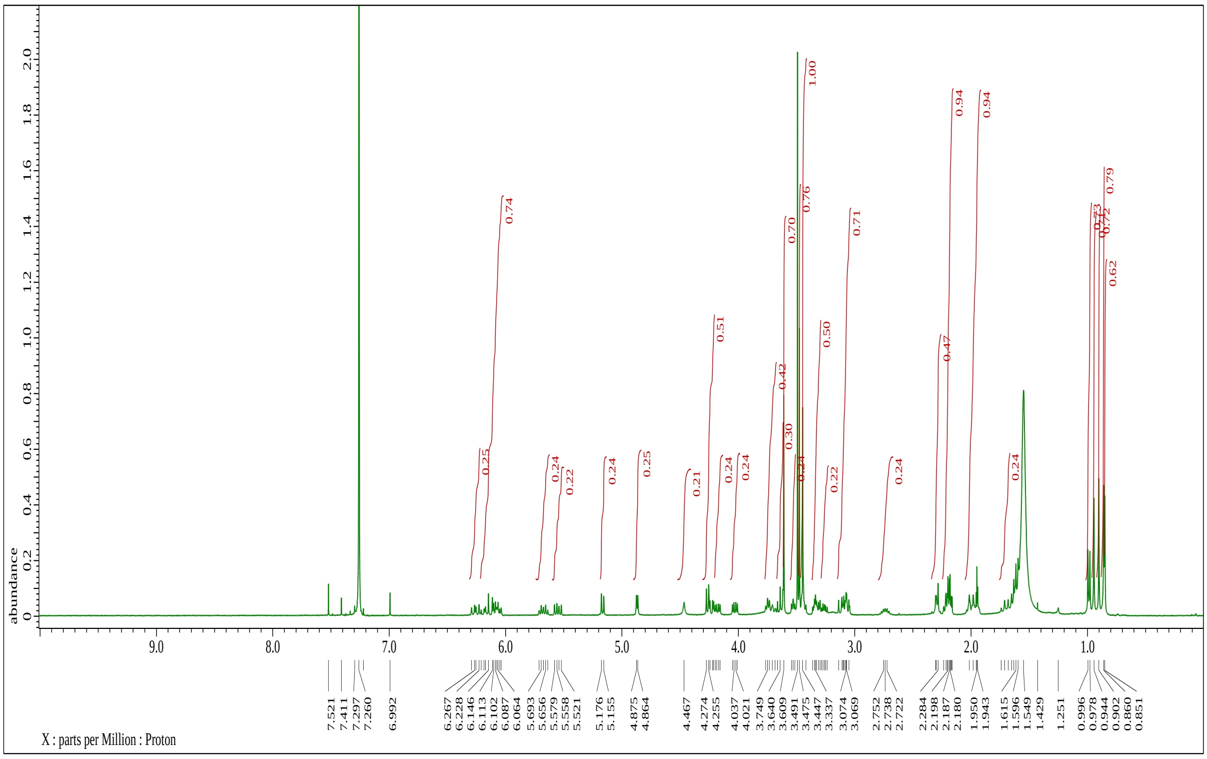

$^{13}C\{^{1}H\}$ NMR (100 MHz, $CDCl_3$) spectrum of polycavernoside E (**1**)

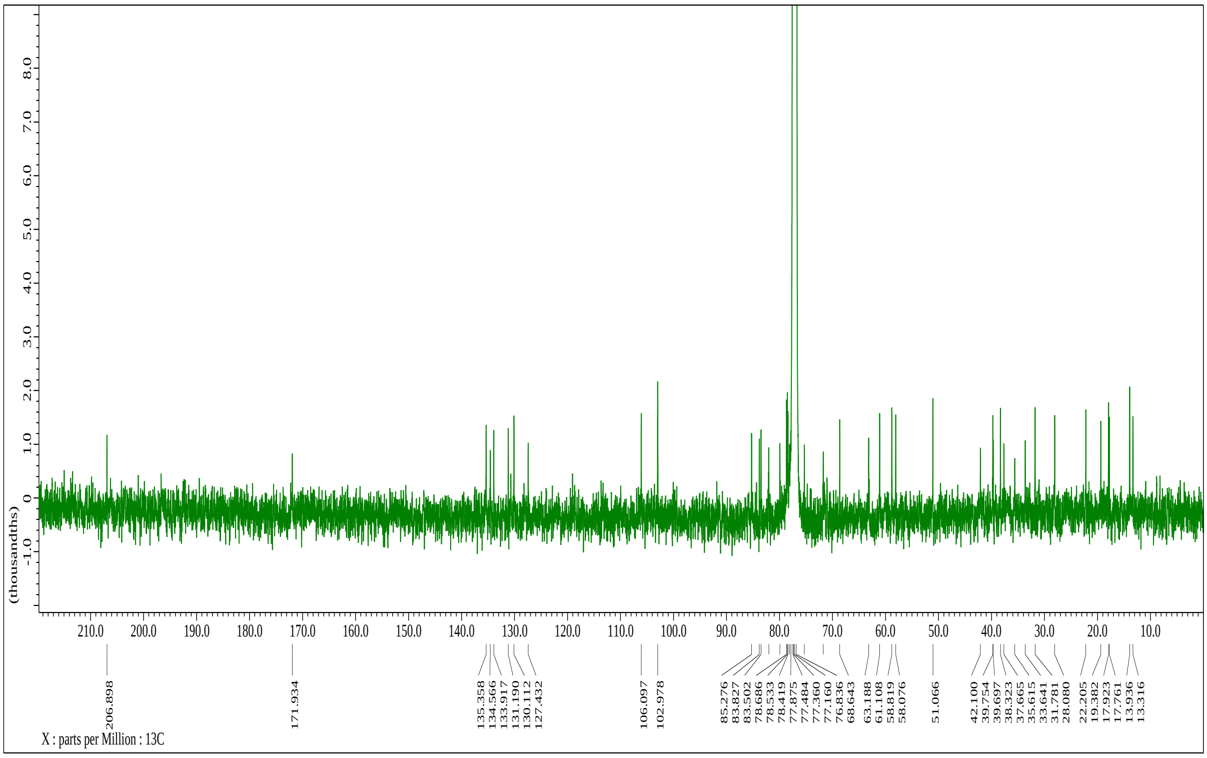

COSY (400 MHz, $CDCl_3$) spectrum of polycavernoside E (**1**)

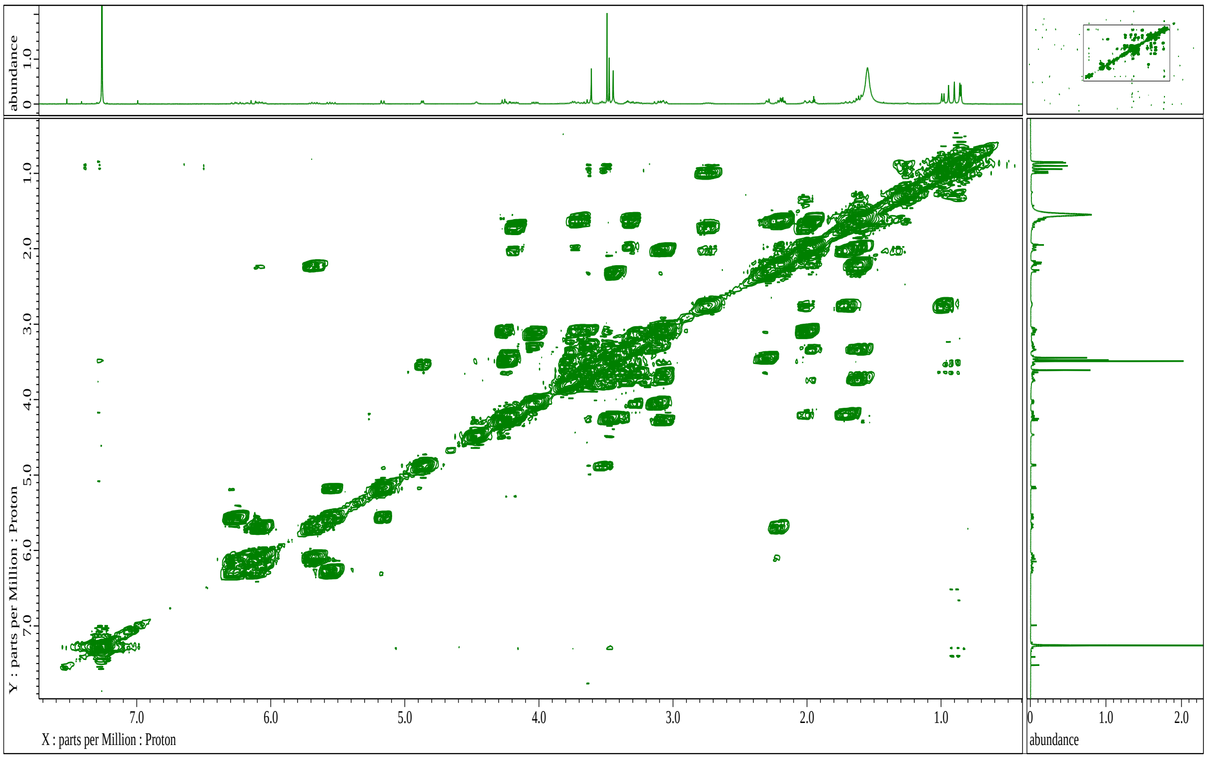

HMQC (400 MHz, $CDCl_3$) spectrum of polycavernoside E (**1**)

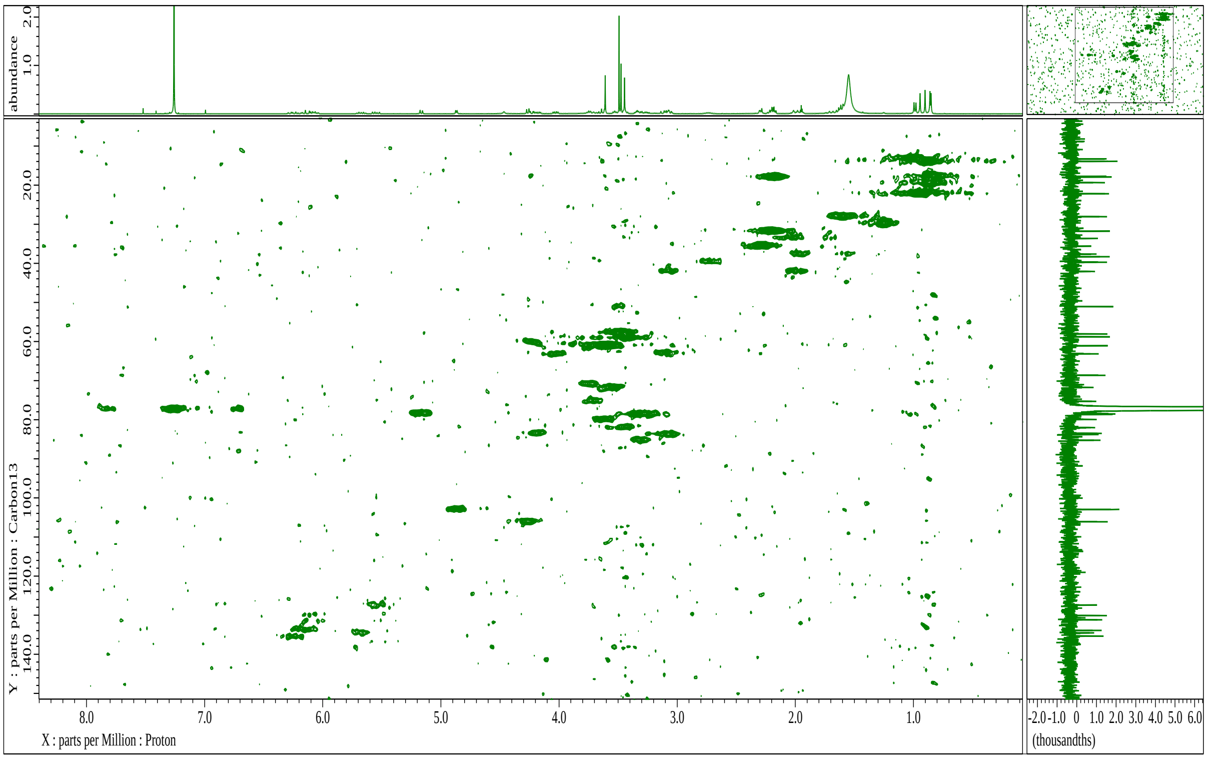

HMBC (400 MHz, $CDCl_3$) spectrum of polycavernoside E (**1**)

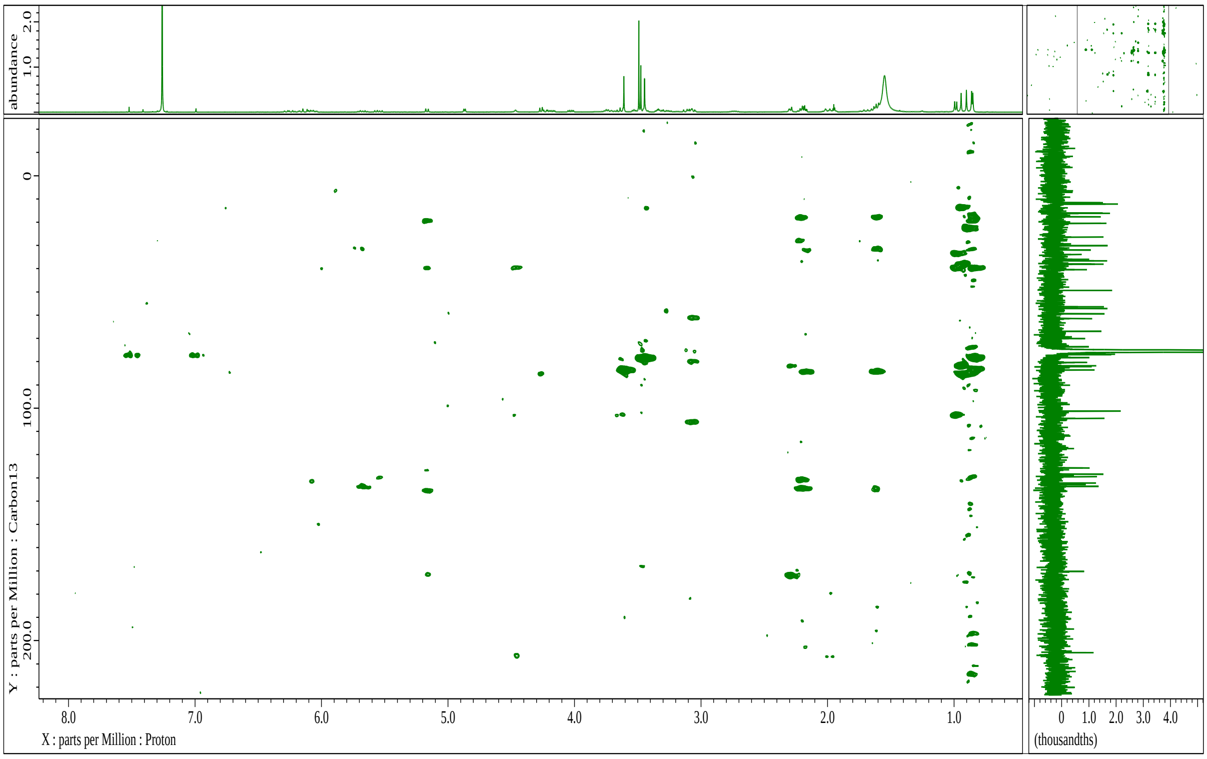

NOESY (400 MHz, $CDCl_3$) spectrum of polycavernoside E (**1**)

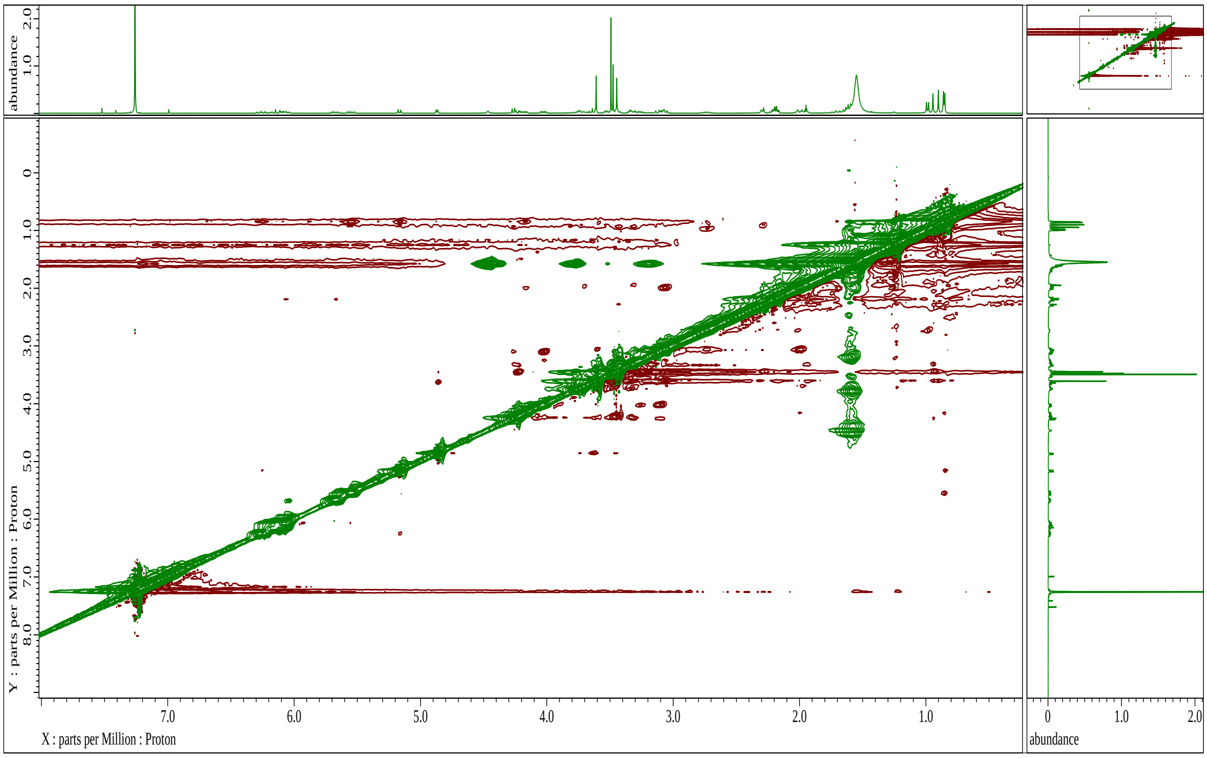

^{1}H NMR (400 MHz, CD_3OD) spectrum of polycavernoside E (**1**)

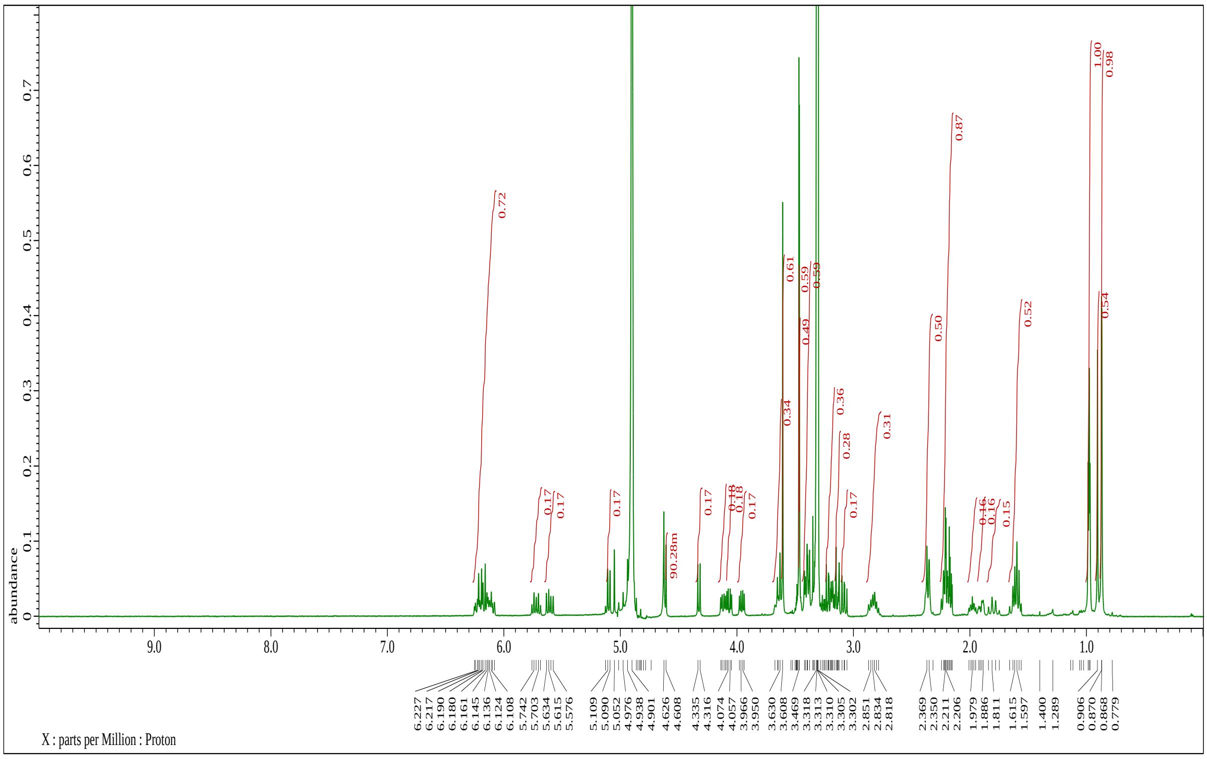

$^{13}C\{^{1}H\}$ NMR (100 MHz, CD_3OD) spectrum of polycavernoside E (**1**)

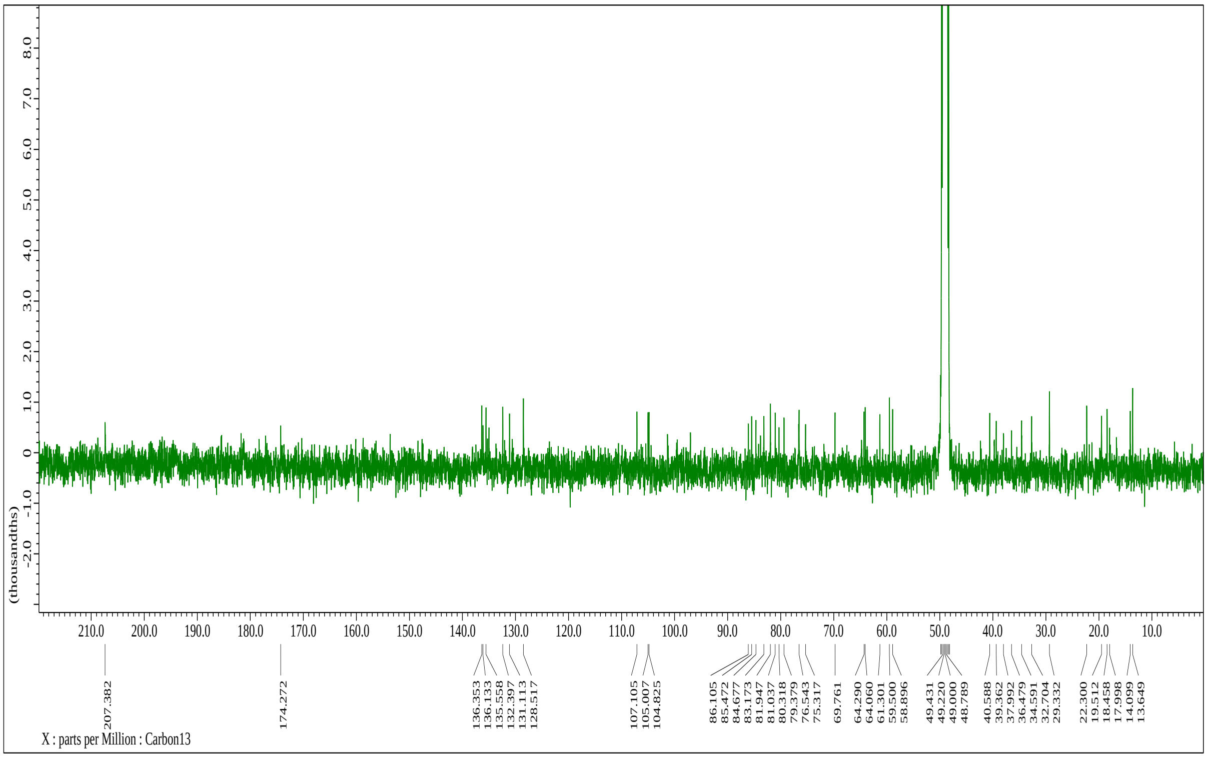

COSY (400 MHz, CD_3OD) spectrum of polycavernoside E (**1**)

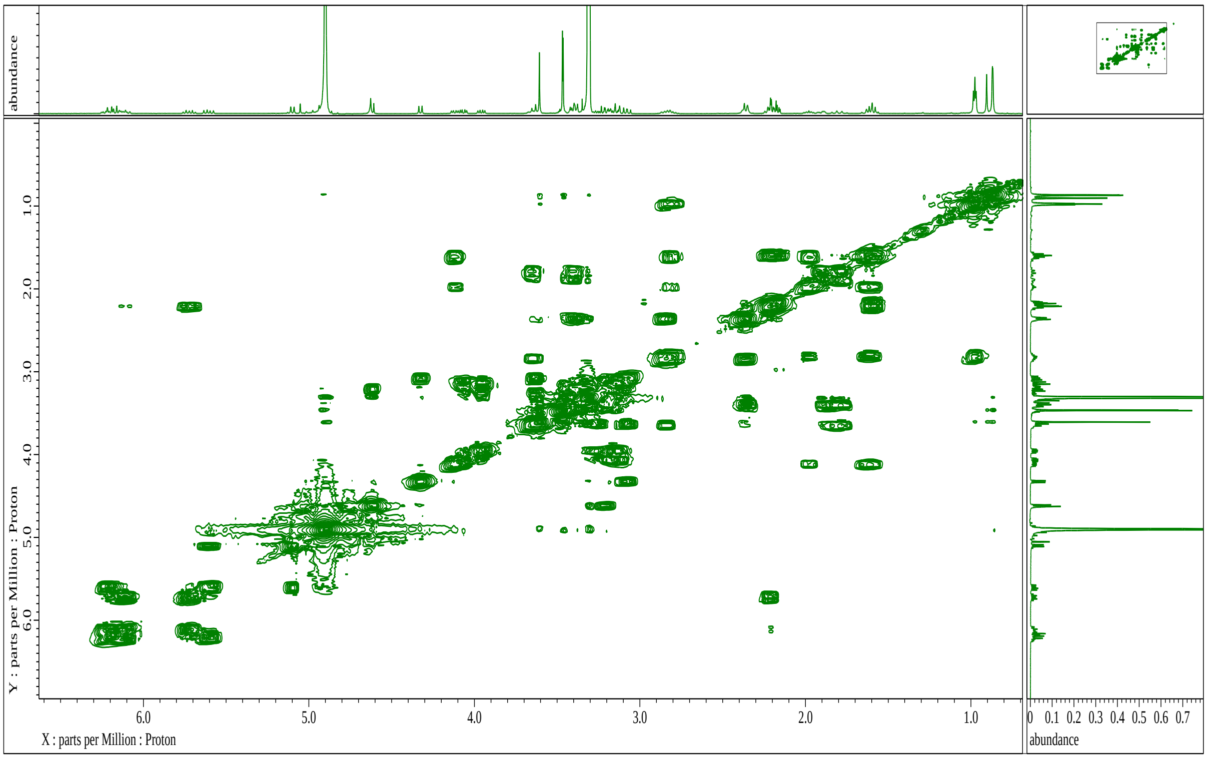

HMQC (400 MHz, CD_3OD) spectrum of polycavernoside E (**1**)

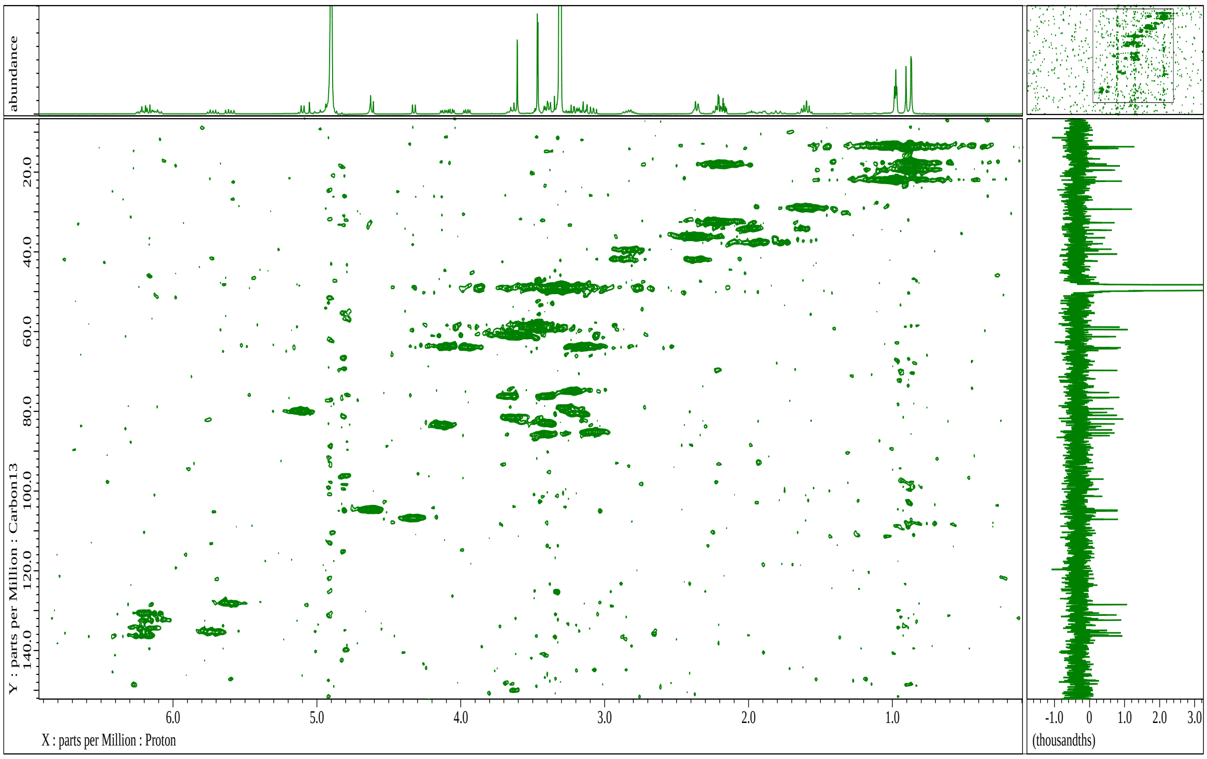

HMBC (400 MHz, CD_3OD) spectrum of polycavernoside E (**1**)

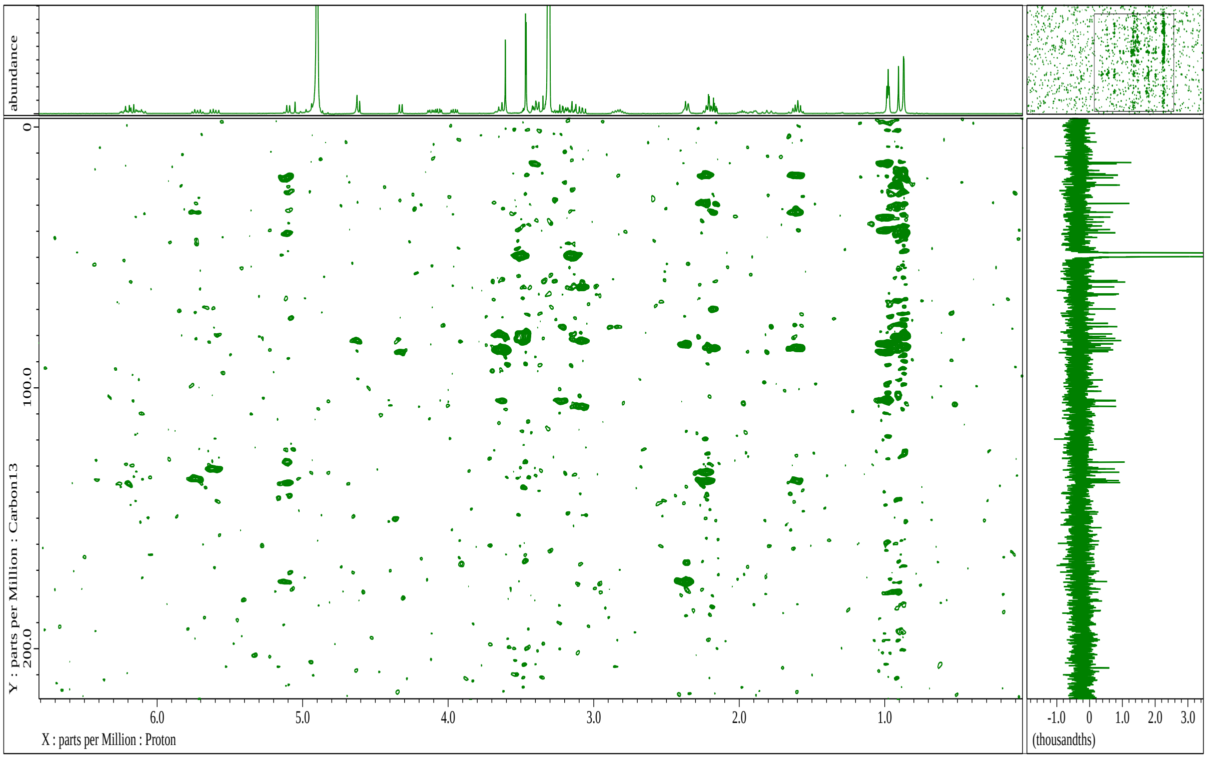

NOESY (400 MHz, CD_3OD) spectrum of polycavernoside E (**1**)

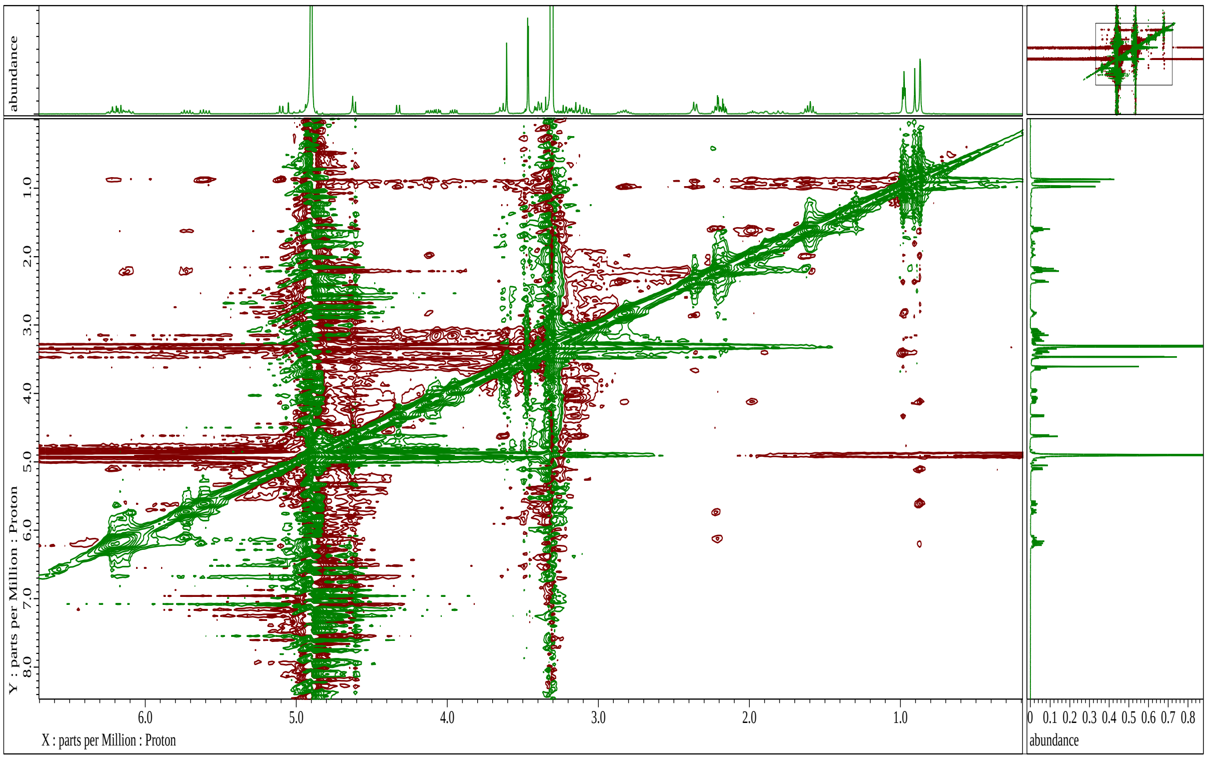

Supplement: File 1 — NMR data for polycavernoside E (1). [file Beilstein_J_Org_Chem-20-645-s001.pdf]
